# Acute Hyperglycaemia, Insulin Resistance, and Cytokine Dysregulation as Predictors of Disease Severity in Non-Diabetic Hospitalised COVID-19 Patients

**DOI:** 10.3390/ijms27052292

**Published:** 2026-02-28

**Authors:** Barbara Grubišić, Luka Švitek, Mihaela Zlosa, Petra Smajić, David Matić, Kristina Kralik, Anita Matić, Marija Santini, Ines Bilić-Ćurčić

**Affiliations:** 1 Department of Infectious Diseases, University Hospital Centre Osijek, 4 Josip Huttler Street, HR-31000 Osijek, Croatia; barbara.grubisic@kbco.hr (B.G.); svitek.luka@kbco.hr (L.Š.); mihaela.zlosa@kbco.hr (M.Z.); petra.smajic@kbco.hr (P.S.); 2 Faculty of Medicine Osijek, Josip Juraj Strossmayer University of Osijek, 4 Josip Huttler Street, HR-31000 Osijek, Croatia; 3 Department of Orthopedics and Traumatology, University Hospital Centre Osijek, 4 Josip Huttler Street, HR-31000 Osijek, Croatia; david.matic@kbco.hr; 4 Department of Medical Statistics and Medical Informatics, Faculty of Medicine, Josip Juraj Strossmayer University of Osijek, 4 Josip Huttler Street, HR-31000 Osijek, Croatia; kristina.kralik@mefos.hr; 5 Department of Pathophysiology, Physiology and Immunology, Faculty of Dental Medicine and Health Osijek, Josip Juraj Strossmayer University of Osijek, 21 Crkvena Street, HR-31000 Osijek, Croatia; anita.matic@fdmz.hr; 6 Department for Infections in Immunocompromised, University Hospital for Infectious Diseases “Dr. Fran Mihaljević”, 16 Mirogojska Street, HR-10000 Zagreb, Croatia; msantini@bfm.hr; 7 Department of Infectious Diseases, School of Medicine, University of Zagreb, 3 Šalata, HR-10000 Zagreb, Croatia; 8 Department for Pharmacology, Faculty of Medicine Osijek, Josip Juraj Strossmayer University of Osijek, 4 Josip Huttler Street, HR-31000 Osijek, Croatia; 9 Department of Endocrinology and Metabolism Disorders, Internal Medicine Clinic, University Hospital Centre Osijek, 4 Josip Huttler Street, HR-31000 Osijek, Croatia

**Keywords:** COVID-19, SARS-CoV-2, diabetes, cytokine dysregulation, newly diagnosed diabetes, hyperglycaemia, insulin resistance

## Abstract

Acute hyperglycaemia is a common COVID-19 complication linked to adverse outcomes. The combined prognostic value of cytokine activation and acute insulin resistance in non-diabetic patients remains unclear. In this prospective cohort study, we enrolled 144 hospitalised adults with RT-PCR-confirmed SARS-CoV-2 infection and no prior diabetes. We aimed to characterise metabolic–inflammatory phenotypes and evaluate their association with disease severity and post-discharge glycaemic outcomes. Patients were classified as normoglycaemic or dysglycaemic based on repeated glucose profiles. Dysglycaemic patients were further phenotyped as stress hyperglycaemia (SHG) or newly diagnosed diabetes (NOD). This classification was based on post-discharge glycaemic assessment at 3 and 6 months, distinguishing transient from persistent hyperglycaemia. Admission hyperglycaemia was associated with a consistently elevated pro-inflammatory cytokine pattern. However, cytokine concentrations were comparable between stress hyperglycaemia and newly diagnosed diabetes, indicating that inflammatory burden alone does not explain metabolic persistence. In contrast, insulin resistance (HOMA-IR) was markedly higher in the newly diagnosed diabetes phenotype. Along with admission oxygenation and key cytokine signals, this contributed to risk stratification for severe disease. In conclusion, early admission assessment of glucose and insulin resistance identifies high-risk metabolic phenotypes. This enables targeted in-hospital risk stratification and post-discharge glycaemic surveillance.

## 1. Introduction

Acute disturbances in glucose regulation, particularly hyperglycaemia, have been identified as significant determinants of outcomes in patients hospitalised with COVID-19 [1]. Early observations during the pandemic indicated that individuals with pre-existing diabetes faced considerably higher risks of severe disease, respiratory failure, and mortality [2]. However, it has also become increasingly clear that even patients without prior diabetes frequently exhibit significant hyperglycaemia during SARS-CoV-2 infection, suggesting that COVID-19 itself may lead to metabolic disturbances [3]. This phenomenon, often termed “stress hyperglycaemia”, affects approximately 50% of hospitalised patients and has been consistently linked with higher mortality and longer hospitalisation across several cohorts [4,5,6,7].

The pathophysiology of dysglycaemia in COVID-19 is multifactorial, involving direct viral entry into pancreatic β-cells, ACE2 downregulation, and endothelial injury, which collectively disrupt insulin secretion and sensitivity [8,9,10,11]. This metabolic instability is further compounded by the systemic stress response, characterised by the release of counter-regulatory hormones such as cortisol, and the frequent administration of corticosteroids for therapeutic purposes [9,12]. Consequently, hyperglycaemia in COVID-19 is not only an outcome of critical illness but also reflects profound metabolic injury with significant prognostic value, consistently serving as a robust indicator of adverse clinical outcomes [2,13,14].

This metabolic dysregulation is intrinsically linked to the systemic inflammatory response, commonly referred to as a “cytokine storm”. As demonstrated by Sardu et al., elevated blood glucose predicts adverse outcomes by augmenting pro-inflammatory signalling, which is associated with elevated IL-6 concentrations and increased disease severity [7]. At the molecular level, elevated concentrations of IL-6, IL-8, and TNF-α interfere with insulin receptor signalling and promote hepatic gluconeogenesis [15,16]. This creates a vicious cycle in which inflammation drives metabolic dysregulation, and hyperglycaemia, in turn, exacerbates the inflammatory cascade and endothelial injury [1,17,18,19,20].

Within this feed-forward loop, acute insulin resistance is a key link through which cytokine-driven inflammation translates into clinically relevant dysglycaemia [15,16]. The Homeostatic Model Assessment for Insulin Resistance (HOMA-IR) is a simple, clinically applicable measure that may help separate the impact of inflammatory burden from underlying metabolic vulnerability [21,22].

Despite the high prevalence of acute hyperglycaemia, its specific manifestations in non-diabetic patients remain poorly understood. Whilst recent studies attribute this metabolic dysregulation to mechanisms such as transient beta cell dysfunction or adiposity-related insulin resistance, the precise phenotypes in this population have not yet been fully defined [4,23]. Many previous studies were limited by the inclusion of patients with pre-existing diabetes or by a lack of strict glucose thresholds to distinguish transient stress hyperglycaemia from new-onset disease [13].

Moreover, existing analyses have often focused on either glucose-based metrics or cytokine patterns in isolation [2,7,16,19]. Crucially, the interplay between specific inflammatory phenotypes (cytokine patterns) and the degree of acute insulin resistance (HOMA-IR) as combined predictors of respiratory failure remains under-investigated in non-diabetic cohorts.

Unlike prior studies, we prospectively restricted the cohort to patients without pre-existing diabetes, adjudicated stress hyperglycaemia versus newly diagnosed diabetes using post-discharge follow-up, and integrated cytokine profiling with admission HOMA-IR to define mechanistically informed metabolic–inflammatory phenotypes and their relationship to COVID-19 severity.

Therefore, the objective of this study was to characterise metabolic–inflammatory phenotypes in COVID-19 patients without pre-existing diabetes by distinguishing stress hyperglycaemia from newly diagnosed diabetes using longitudinal glycaemic assessment, and to evaluate their associations with cytokine activation, insulin resistance (HOMA-IR), and clinical severity.

## 2. Results

### 2.1. Glycaemic Classification and Insulin Resistance

Based on glycometabolic assessment, the dysglycaemic cohort (n=72) was categorised into two subgroups: 43 patients (59.7%) were classified as having stress hyperglycaemia, whilst 29 (40.3%) met the ADA criteria for newly diagnosed diabetes. Despite a marked disparity in admission fasting glucose concentrations between the newly diagnosed diabetes and stress hyperglycaemia subgroups (median 11.8 [IQR 9.6–14.3] vs. 8.1 [7.9–8.5] mmol/L; p<0.001), HbA1c values did not differ significantly between these phenotypes (5.5% [5.1–6.3] vs. 5.1% [4.2–6.2]; p=0.12). This finding highlights the acute nature of glycaemic dysregulation, driven by infection-induced inflammatory response rather than by pre-existing metabolic dysfunction.

Assessment of insulin resistance revealed a clear dose–response pattern, with stepwise increases in HOMA-IR across normoglycaemia, stress hyperglycaemia, and newly diagnosed diabetes, paralleling progressively greater respiratory impairment and clinical severity. Normoglycaemic patients maintained a median HOMA-IR of 1.9, whereas the dysglycaemic cohort demonstrated a marked increase to 6.8 (p<0.001). As detailed in Table 1, insulin resistance was most pronounced in newly diagnosed diabetes (median HOMA-IR 11.2) compared with stress hyperglycaemia (median 5.6; p<0.001).

**Figure 1:**
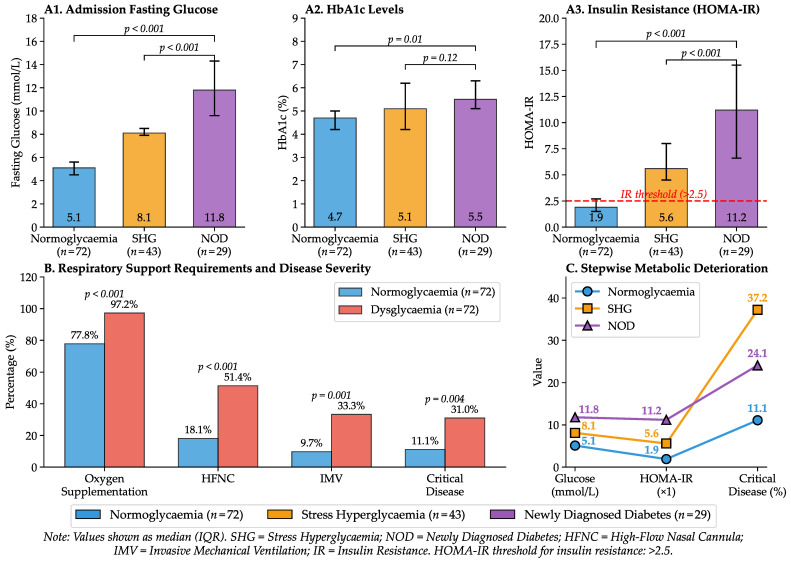
Glycaemic Classification, Insulin Resistance, and Clinical Outcomes.

The increase in insulin resistance corresponded with clinical severity and respiratory impairment. Patients with dysglycaemia required oxygen supplementation significantly more frequently than normoglycaemic controls (97.2% vs. 77.8%; p<0.001). Furthermore, the dysglycaemic group exhibited markedly higher dependency on advanced respiratory support, including high-flow nasal cannula (51.4% vs. 18.1%; p<0.001) and invasive mechanical ventilation (33.3% vs. 9.7%; p=0.001). Consequently, the rate of critical disease was nearly threefold higher in the dysglycaemic group (30.6% vs. 11.1%; p=0.004). Higher HOMA-IR values were observed alongside more frequent oxygen requirement and greater use of advanced respiratory support. These relationships between glycaemic classification, insulin resistance, and clinical outcomes are illustrated in Figure 1.

### 2.2. Cytokine Profiling and Correlation with Disease Severity

Multiplex immunoassay analysis of six key cytokines (IL-6, IL-2, IL-4, IL-8, IFN-γ, and TNF-α) revealed an extensive pro-inflammatory profile in patients with admission hyperglycaemia. Compared with normoglycaemic controls, these patients exhibited significantly elevated median concentrations of IL-6, IL-8, TNF-α, IFN-γ, and IL-2. Admission metabolic markers and cytokine concentrations, including both the normoglycaemia versus dysglycaemia comparison and the SHG versus NOD comparison, are summarised in Table 1. Importantly, the magnitude of cytokine activation was comparable between SHG and NOD, with similar elevations in IL-6, IL-8/CXCL8, and TNF-α in both phenotypes. Notably, this dissociation, along with similar cytokine activation in SHG and NOD despite markedly different insulin resistance, indicates that inflammatory burden alone does not distinguish transient stress hyperglycaemia from a persistent dysglycaemic phenotype.

**Figure 2:**
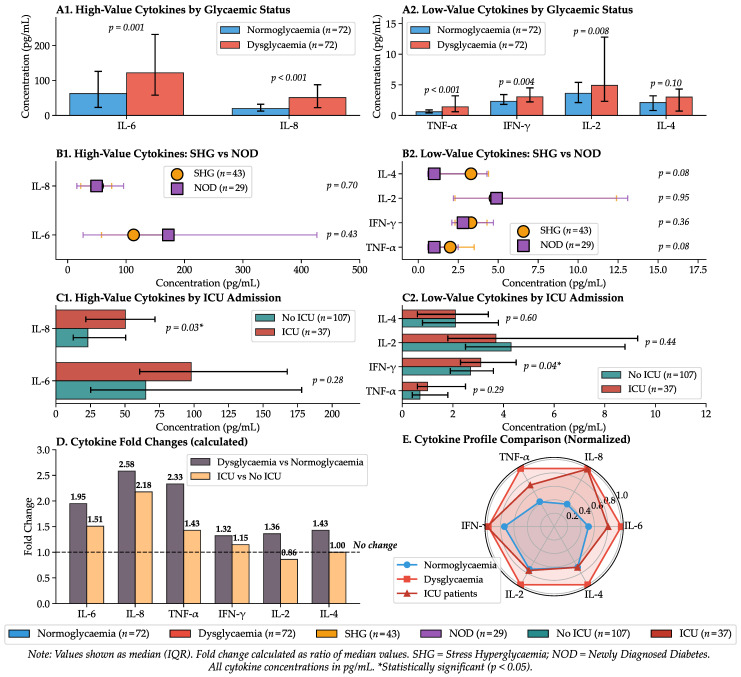
Cytokine Profiling and Correlation with Disease Severity.

This cytokine dysregulation served as a strong predictor of adverse outcomes. Non-survivors had significantly higher admission IL-6 and IL-8/CXCL8 concentrations than survivors (Table 2). Patients requiring ICU admission exhibited higher IL-8 and IFN-γ than those managed on the general ward (Table A1). Regarding metabolic status, TNF-α (AUC 0.757) and IL-8 (AUC 0.739) demonstrated superior predictive ability for stress hyperglycaemia compared with IL-6. Furthermore, logistic regression analysis identified admission concentrations of IL-6, IL-8, and TNF-α, alongside fasting glucose, HbA1c, insulin, and HOMA-IR, as significant predictors of persistent hyperglycaemia or diabetes at 6-month follow-up. The cytokine profiling results, including the correlation between inflammatory markers and disease severity across glycaemic phenotypes, are comprehensively illustrated in Figure 2.

Compared with normoglycaemic controls, dysglycaemic patients had higher admission cytokine concentrations, whereas within the dysglycaemic cohort, cytokine levels were similar between SHG and NOD (Table 1).

### 2.3. Acute Clinical Outcomes and Mortality

**Figure 3:**
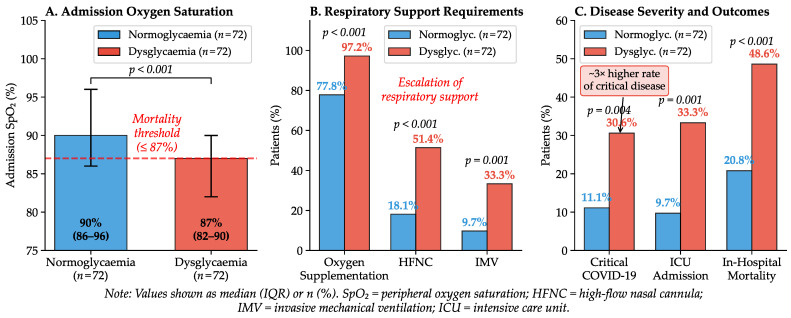
Admission Oxygenation, Respiratory Support, Severity, and Outcomes.

Admission oxygen saturation was significantly compromised in patients with hyperglycaemia compared with the normoglycaemic group (median 87% vs. 90%; p<0.001). Figure 3 and Table A2 present a comprehensive comparison of radiological findings, oxygenation parameters, respiratory support requirements, and disease severity across glycaemic groups. In this cohort, admission SpO_2_ was lower in non-survivors and ICU patients than in survivors and non-ICU patients (86% vs. 90%; p<0.001). Admission SpO_2_ independently predicted ICU admission (OR 0.92; p=0.03) and in-hospital mortality (OR 0.92; p=0.004) in multivariable models. ROC analysis identified ≤87% as the optimal admission SpO_2_ threshold for mortality risk stratification (AUC 0.685; Table 3).

**Figure 4:**
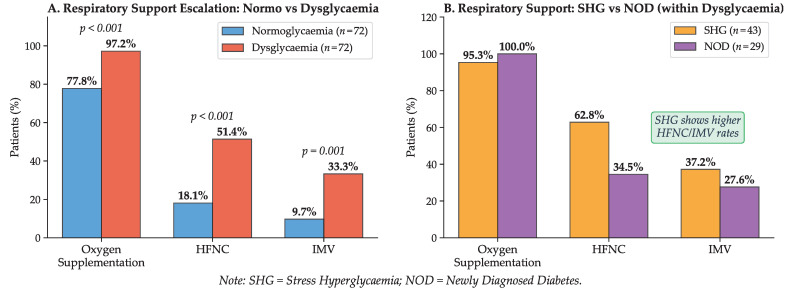

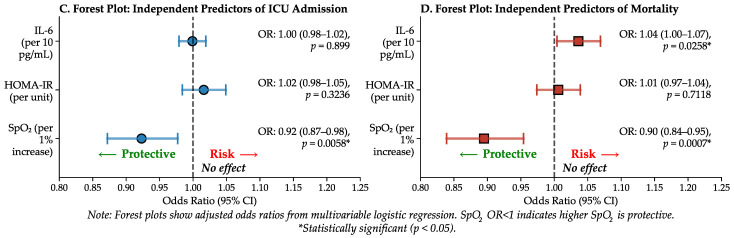
Escalation of Respiratory Support and Outcomes.

Figure 3 illustrates the marked disparity in oxygenation status and respiratory support requirements between normoglycaemic and dysglycaemic patients. Dysglycaemic patients had significantly higher requirements for supplemental oxygen (97.2% vs. 77.8%), high-flow nasal cannula (51.4% vs. 18.1%), and mechanical ventilation (33.3% vs. 9.7%). The stepwise escalation of respiratory support, along with the corresponding odds ratios for adverse outcomes, is presented in the forest plot shown in Figure 4.

Mortality was higher among dysglycaemic patients, affecting 53.5% of the stress hyperglycaemia group and 41.4% of those with newly diagnosed diabetes, compared with 20.8% in normoglycaemic controls. Although mortality was numerically higher in SHG than in NOD, this difference did not reach statistical significance. Overall, admission dysglycaemia was associated with greater care escalation and disease severity.

Exploratory analyses of selected non-glycaemic biomarkers in relation to survival are provided in Table A3.

Survivors with dysglycaemia were followed prospectively for six months post-discharge. In the stress hyperglycaemia group, glucose concentrations normalised in most patients; the prevalence of isolated hyperglycaemia declined from 17% at discharge to 2% at six months.

In contrast, the prevalence of diabetes remained largely unchanged at six-month follow-up among those initially diagnosed with newly diagnosed diabetes, indicating that hyperglycaemia in this subgroup predominantly reflected permanent metabolic dysfunction rather than a transient stress response.

## 3. Discussion

In this prospective cohort of hospitalised COVID-19 patients without pre-existing diabetes, admission dysglycaemia was associated with poorer respiratory status and a more severe clinical course. This association was observed alongside heightened inflammatory signalling, supporting a close linkage between early metabolic disturbance and systemic inflammation. These results are consistent with prior studies that have identified dysglycaemia as an early indicator of clinical deterioration and adverse outcomes [4,24].

Most prior studies have evaluated either admission glucose as a prognostic marker or inflammatory profiles in isolation, often without longitudinal adjudication of glycaemic phenotype. By integrating cytokine profiling with admission insulin resistance and post-discharge glycaemic reassessment, our study links acute metabolic–inflammatory signatures to both in-hospital severity and persistence of dysglycaemia.

Dysglycaemia occurred in the context of a pronounced inflammatory response, with elevated pro-inflammatory signalling (including IL-6, IL-8, and TNF-α), supporting the concept that acute metabolic destabilisation in COVID-19 is tightly coupled to systemic inflammation. In parallel, dysglycaemic patients exhibited markedly higher insulin resistance, with HOMA-IR values three to five times higher than normoglycaemic controls, consistent with the established capacity of IL-6 and TNF-α to disrupt insulin signalling and promote both peripheral and hepatic insulin resistance [14,25,26]. This is also in line with prior observations of functional β-cell impairment and acute glycometabolic disruption in COVID-19 [4].

A key finding of this study is the dissociation between inflammatory activation and insulin resistance: cytokine concentrations were comparable between stress hyperglycaemia and newly diagnosed diabetes, whereas insulin resistance differed markedly, indicating that acute insulin resistance—rather than inflammatory burden alone—was the stronger discriminator of the persistent dysglycaemic phenotype. Collectively, this dissociation represents the central conceptual contribution of our study and refines the prevailing assumption that inflammatory activation alone explains metabolic persistence.

The combination of inflammation, glucotoxicity, and insulin resistance likely contributes to rapid clinical deterioration. In multivariable models, admission IL-6 and selected metabolic and oxygenation markers remained independent statistical predictors of ICU admission and mortality [27]. However, given the observational design, these associations should not be interpreted as evidence of causality. Accordingly, prognostication may benefit from joint assessment of metabolic and inflammatory markers [23].

Our observation that non-survivors exhibited markedly elevated baseline IL-6 and IL-8 concentrations aligns with the findings of Del Valle et al., thereby underscoring the utility of these biomarkers for early risk stratification [19]. Importantly, however, our data demonstrate that in non-diabetic patients, the intensity of this cytokine response correlates closely with the severity of hyperglycaemia. This suggests that acute dysglycaemia is not merely a secondary feature, but rather a clinically relevant marker of a high-risk metabolic–inflammatory state that co-occurs with more severe inflammation and poorer outcomes.

From a clinical perspective, SpO_2_ and admission glucose remain simple frontline markers for early risk assessment. Cytokine profiling is not intended to replace these measures, but may provide complementary biological context on the host inflammatory response and refine stratification when integrated with metabolic indices in settings where such testing is available.

Clinically, these findings support early metabolic phenotyping at admission. Patients with marked insulin resistance may warrant intensified inpatient glucose monitoring and a lower threshold for structured post-discharge reassessment, even when admission HbA1c is below diagnostic thresholds. Where available, cytokine profiling may provide supportive prognostic information on the host inflammatory response, but it should be interpreted as complementary rather than a discriminator of SHG versus NOD. A key strength of this study is the longitudinal adjudication of stress hyperglycaemia versus newly diagnosed diabetes using post-discharge glycaemic assessment, which reduces misclassification inherent to admission-only definitions and links acute phenotypes to clinically relevant persistence of dysglycaemia. In this context, pronounced admission insulin resistance provides a practical signal for identifying patients who may benefit from structured post-discharge glycaemic surveillance [4,28].

Metabolic dysfunction was closely associated with a more severe clinical course. Dysglycaemic patients required significantly longer hospitalisation and escalated respiratory care compared with normoglycaemic controls. Notably, oxygen dependency was nearly universal (97%) in this group, whilst the need for invasive mechanical ventilation tripled. These data support a robust association between acute hyperglycaemia and more severe pulmonary dysfunction, a finding well documented in prior research [4,13,14,28,29,30].

Regarding fatal outcomes, admission hyperglycaemia emerged as a critical prognostic factor, even in patients without known diabetes. Consistent with Wang et al. [13], we observed significantly higher mortality among dysglycaemic patients than among normoglycaemic controls. This risk was elevated in both the stress hyperglycaemia and newly diagnosed diabetes subgroups, indicating that the severity of acute glucose elevation, rather than the specific phenotype, is associated with immediate survival outcomes. These findings are consistent with a close coupling between acute hyperglycaemia, inflammatory activation, and clinical deterioration in fatal cases, although the present design does not permit causal inference regarding the direction of these relationships.

A median HbA1c of 5.5% was a key characteristic of our newly diagnosed diabetes group. This differs substantially from previous studies, in which values were typically higher than the 6.5% diagnostic threshold [20,28]. Despite admission HbA1c values falling below the diabetic threshold, the diagnosis was confirmed by sustained hyperglycaemia at 3- and 6-month follow-up. This mirrors the observations of Montefusco et al., who distinguished “new-onset” diabetes from transient stress hyperglycaemia by the persistence of glycometabolic dysregulation in patients with no prior history of disease [4]. Notably, our regression analysis identified TNF-α as a significant predictor of this outcome, suggesting that acute inflammatory stress served as a triggering factor, unmasking latent beta cell dysfunction in susceptible individuals. This supports the hypothesis proposed by Rubino et al. that SARS-CoV-2 can induce true newly diagnosed diabetes rather than merely unmasking latent disease [24]. However, our study cannot distinguish whether the observed phenotype reflects de novo diabetes, unmasking of latent disease, or both. These findings suggest that early recognition of insulin resistance may justify prompt glycaemic intervention, judicious use of corticosteroids, and structured post-discharge metabolic follow-up in high-risk COVID-19 patients.

It should be acknowledged that the present cohort consisted exclusively of hospitalised patients, representing a population with inherently more severe clinical presentations of COVID-19. Consequently, the high prevalence of respiratory failure, oxygen dependency, and adverse outcomes observed in our study reflects not only metabolic dysregulation but also the underlying severity of acute illness requiring hospitalisation. However, significant variations in insulin resistance and the inflammatory response within this uniformly high-risk population highlight the independent association between glycometabolic disturbances and markers of disease severity within this hospitalised high-risk population.

Notably, mortality was numerically higher in the stress hyperglycaemia subgroup (53.5%) than in newly diagnosed diabetes (41.4%), despite the latter exhibiting more severe insulin resistance. This observation may reflect the acute severity of the inflammatory insult in stress hyperglycaemia or differences in host vulnerability, although the comparison did not reach statistical significance and warrants further investigation in larger cohorts.

We also observed a lower vaccination rate in the dysglycaemic group; although not statistically significant, this finding may reflect residual confounding (healthcare access or health behaviours) and should be interpreted as hypothesis-generating.

Finally, exploratory analyses suggested that lower admission 25(OH)D levels were more frequent in non-survivors; this observation was not a prespecified objective and should be interpreted as hypothesis-generating. Nonetheless, the direction of association is consistent with prior reports linking vitamin D deficiency to poorer COVID-19 outcomes [31,32,33].

## 4. Materials and Methods

### 4.1. Study Design and Objectives

This prospective cohort study was conducted at the Department of Infectious Diseases at the Clinical Hospital Centre Osijek (Osijek, Croatia). Consecutive eligible adults were enrolled between January 2023 and December 2024. SARS-CoV-2 whole-genome sequencing was not performed for study samples, and individual-level variant assignment was therefore not available. Based on ECDC genomic surveillance for the EU/EEA, the enrolment period coincided with Omicron sublineage predominance, including the emergence and expansion of XBB-related lineages during 2023 and the dominance of BA.2.86-descendant lineages (including JN.1) during 2024 [34,35].

The primary objective was to determine whether admission hyperglycaemia, stratified as stress hyperglycaemia or newly diagnosed diabetes, is associated with a distinct cytokine profile and whether these metabolic–inflammatory phenotypes predict a severe clinical course.

The pre-specified primary endpoint was a severe clinical course, defined as ICU admission and/or in-hospital mortality. Key secondary endpoints included escalation of respiratory support, length of hospital stay, and persistence of dysglycaemia or incident diabetes at 3 and 6 months post-discharge. The study received ethical approval from the Clinical Hospital Centre Osijek Ethics Committee and the Ethics Committee of the University of Osijek School of Medicine.

### 4.2. Participants

A total of 144 hospitalised adults (≥18 years) with confirmed SARS-CoV-2 infection and no prior history of diabetes were enrolled in this study. A COVID-19 diagnosis was confirmed by a positive RT-PCR test result. Patients who died within the first 24 h of admission, those with pre-existing diabetes, pregnant women, and individuals lacking adequate clinical documentation were excluded.

Participants were stratified into a normoglycaemic group (n=72) and a dysglycaemic group (n=72) based on repeated fasting glucose measurements and daily glycaemic profiles obtained throughout hospitalisation. Within the dysglycaemic cohort, patients were further categorised as having stress hyperglycaemia or newly diagnosed diabetes.

The definitive distinction between stress hyperglycaemia and newly diagnosed diabetes within the dysglycaemic population was determined retrospectively, based on glycaemic status at one-, three-, and six-month follow-up assessments. Stress hyperglycaemia was defined as transient in-hospital hyperglycaemia with subsequent normalisation of glucose concentrations during the follow-up period. Conversely, newly diagnosed diabetes was defined as persistent hyperglycaemia meeting American Diabetes Association (ADA) diagnostic criteria [36] at 3 months post-discharge, regardless of lower initial HbA1c values at admission. This approach was employed to minimise misclassification bias whilst acknowledging the potential for residual misclassification in deceased patients.

For patients who died prior to follow-up assessment, glycaemic phenotype was assigned based on persistent severe fasting hyperglycaemia (≥11.1 mmol/L on admission and on at least 2 consecutive inpatient measurements), in accordance with ADA diagnostic thresholds.

Baseline demographic characteristics and comorbidities are summarised in Table 4 to characterise the cohort.

### 4.3. Methods

The clinical evaluation included a structured medical history (age, sex, day of illness at admission, comorbidities, vaccination status, and presenting COVID-19 symptoms), as well as a comprehensive physical examination with measurements of vital signs (blood pressure, heart rate, oxygen saturation, and respiratory rate). Upon admission, all patients underwent comprehensive laboratory evaluation, including complete and differential blood counts; serum glucose and electrolyte measurements; liver and muscle enzymes (ALT, AST, GGT, ALP, LDH, CK); inflammatory and cardiac biomarkers (CRP, ferritin, PCT, hs-TnI); renal function indicators (urea, creatinine); coagulation parameters (PT, aPTT, fibrinogen, D-dimer, antithrombin III); and vitamin D concentrations. Cytokine concentrations (IFN-γ, IL-2, IL-4, IL-6, IL-8/CXCL8, and TNF-α) were measured using a multiplex immunoassay platform. For analyses using repeat laboratory values, we used the first in-hospital repeat measurement after admission (routine monitoring) and included only results obtained before ICU transfer or death.

The cytokine panel was predetermined to identify unique but complementary immunological mechanisms associated with acute COVID-19 and metabolic stress. It included IL-6, IL-8/CXCL8, and TNF-α as principal mediators of innate pro-inflammatory activity, together with IFN-γ and IL-2 as markers of Th1/T-cell activation and IL-4 reflecting Th2-mediated counter-regulatory signalling. This broader panel enabled assessment of whether adaptive immune polarisation provides additional discriminatory value beyond the existing innate inflammatory signal, particularly regarding insulin resistance and the persistence of dysglycaemia.

Study outcomes included the clinical course of COVID-19 (radiographic findings, development of complications, and need for oxygen supplementation), glycaemic profiles during hospitalisation, and major clinical endpoints such as discharge, transfer to the intensive care unit, or in-hospital mortality.

Clinical outcomes were prospectively recorded during hospitalisation and follow-up and analysed according to the pre-specified endpoints outlined in Section 4.1. Exploratory outcomes included cytokine profiling, correlation analyses between metabolic and inflammatory markers, and ROC-derived cut-off estimation.

Disturbances in glycaemic control were additionally monitored for up to six months following discharge, including the occurrence of persistent hyperglycaemia, resolution of dysglycaemia, or the diagnosis of diabetes. This adjudication strategy follows established methodological precedents aimed at minimising misclassification bias inherent in admission-only definitions during acute infection. Furthermore, admission HOMA-IR was utilised as a sensitive marker of acute insulin resistance to capture metabolic instability during the systemic inflammatory response that may not be reflected by HbA1c alone.

Exploratory analyses included selected non-glycaemic biomarkers (25-hydroxyvitamin D and red cell distribution width) in relation to in-hospital outcomes.

**Figure 5:**
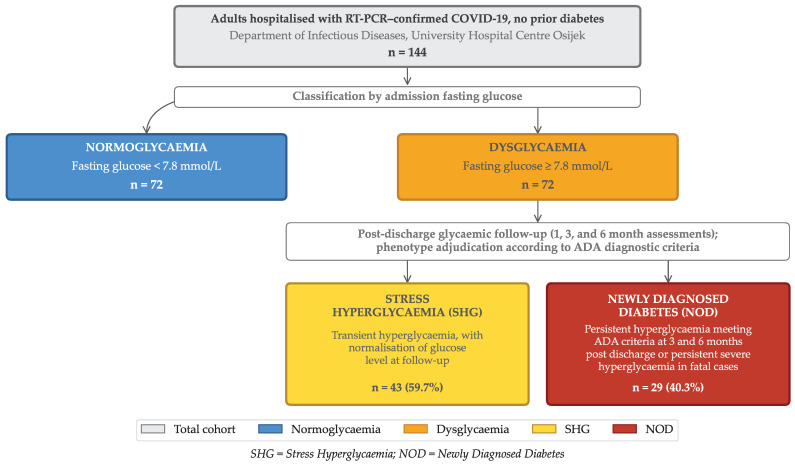
STROBE Flow Diagram of Participant Inclusion, Glycaemic Classification, and Clinical Outcomes.

Participant inclusion, exclusions, and glycaemic phenotyping are summarised in the STROBE flow diagram (Figure 5).

### 4.4. Laboratory Assays

All laboratory analyses were performed at the Department of Clinical Laboratory Diagnostics, University Hospital Centre Osijek (Osijek, Croatia) using automated platforms with manufacturer reagents according to standard procedures (haematology: Sysmex analyser, Sysmex Corporation, Kobe, Japan; biochemistry/electrolytes: AU480, Beckman Coulter, Brea, CA, USA; coagulation: BCS XP, Siemens Healthineers AG, Erlangen, Germany). Procalcitonin (PCT) was measured by electrochemiluminescence immunoassay (ECLIA) on a COBAS e601 analyser (Roche Diagnostics GmbH, Mannheim, Germany), and hs-TnI was determined using a homogeneous LOCI-based immunoassay on a Dimension EXL analyser with LM (Siemens Healthcare Diagnostics, Newark, NJ, USA). HbA1c was measured on the Dimension EXL analyser (Siemens Diagnostics, Munich, Germany), and serum insulin was determined by chemiluminescent immunoassay (CLIA) on the LIAISON XL analyser (DiaSorin S.p.A., Saluggia, Italy); HOMA-IR was calculated as fasting insulin (μIU/mL) × fasting glucose (mmol/L)/22.5. Admission serum cytokines (IL-6, IL-2, IL-4, IL-8/CXCL8, IFN-γ, TNF-α) were quantified using customised ProcartaPlex multiplex assays (eBioscience, Affymetrix by Thermo Fisher Scientific, Waltham, MA, USA) on a Luminex 200 platform, with concentrations calculated using ProcartaPlex Analyst software and expressed as pg/mL.

### 4.5. Sample Size and Statistical Analysis

The minimum sample size required to detect a medium effect size between two independent groups, with a significance level of 0.05 and statistical power of 0.80, was calculated to be at least 128 participants (64 per group) using G*Power software (version 3.1.2).

Categorical variables were summarised as frequencies and percentages, whilst continuous variables were described using means and standard deviations or medians with interquartile ranges, depending on distribution. Normality was assessed using the Shapiro–Wilk test. Group differences were evaluated using the $\chi^{2}$ test or Fisher’s exact test for categorical variables and the Student *t*-test or Mann–Whitney *U* test for continuous variables, as appropriate. Comparisons across more than two independent groups were performed using ANOVA or the Kruskal–Wallis test, followed by a Conover post hoc analysis.

Correlations between metabolic markers (glucose, insulin, HOMA-IR) and inflammatory cytokines were assessed using Pearson or Spearman correlation coefficients, depending on data distribution. Bivariate and multivariable logistic regression models were employed to identify independent predictors of severe COVID-19 outcomes, including oxygen requirement and ICU admission.

Multivariable models were planned to estimate independent predictors of severe outcomes. Given the sample size and event rates, models were intentionally kept parsimonious, with candidate variables selected based on clinical plausibility and bivariate associations to minimise overfitting. To operationally assess potential multicollinearity among candidate metabolic and inflammatory predictors, we screened pairwise correlations (Pearson or Spearman, as appropriate). A predefined threshold of |r|≥0.70 was used to indicate high collinearity; highly correlated variables were not entered simultaneously into the same multivariable model. In such cases, a representative marker was retained based on clinical plausibility and the strength of the bivariate association to preserve model stability.

To assess and mitigate potential multicollinearity among metabolic and inflammatory predictors, we examined pairwise correlations and avoided simultaneously including highly correlated biomarkers within the same model; inflammatory markers were evaluated in a limited/representative manner to preserve model stability. Adjusted odds ratios (ORs) with 95% confidence intervals (CIs) are reported.

Receiver operating characteristic (ROC) curve analyses were performed to determine the discriminative ability of HOMA-IR and selected cytokines for predicting dysglycaemia and adverse clinical outcomes. Optimal cut-off values, sensitivity, specificity, and area under the curve (AUC) were calculated. All tests were two-tailed, and statistical significance was set at α<0.05. Analyses were conducted using MedCalc^®^ Statistical Software version 22.023, IBM SPSS Statistics version 23.0, and Python version 3.12.

## 5. Conclusions

Early admission phenotyping that integrates glucose with insulin resistance provides a practical approach to identify hospitalised COVID-19 patients without known diabetes at high risk for severe disease and persistent dysglycaemia, supporting targeted inpatient management and post-discharge metabolic surveillance. Acute hyperglycaemia in this setting reflects profound metabolic dysregulation associated with pro-inflammatory cytokine activation (IL-6, TNF-α, IL-8) and severe insulin resistance. We identified a phenotype consistent with newly diagnosed diabetes, characterised by marked insulin resistance and linked to persistent metabolic dysfunction at six months.

Whilst cytokine concentrations were comparable between stress hyperglycaemia and newly diagnosed diabetes, selected cytokine signals were associated with adverse outcomes. Accordingly, in this cohort, cytokine profiling primarily added biological and pathophysiological context, whereas admission glucose, HOMA-IR, and oxygenation indices remained the core practical markers for early stratification; cytokine profiling may therefore be used as a supportive prognostic context where available.

### Strengths and Limitations

This study has several notable strengths. It prospectively investigated a well-characterised cohort of hospitalised COVID-19 patients without pre-existing diabetes, allowing for a focused evaluation of acute dysglycaemia independent of known chronic metabolic disease. A key strength is the longitudinal classification of glycaemic phenotypes, which distinguishes stress hyperglycaemia from newly diagnosed diabetes using post-discharge follow-up, thereby overcoming a major limitation of prior studies that relied solely on admission glucose or HbA1c values. Furthermore, the simultaneous assessment of insulin resistance (HOMA-IR) and a comprehensive cytokine profile enabled integrated metabolic–inflammatory phenotyping and provided mechanistic insight into disease severity. The extensive clinical, laboratory, and outcome data, combined with multivariable analyses, strengthen the robustness and clinical relevance of the findings.

Several limitations should be acknowledged. The study was conducted at a single centre, which may limit generalisability to other populations or healthcare settings. Classification of the glycaemic phenotype in patients who died before follow-up relied on inpatient glucose patterns, introducing potential misclassification bias. Pre-infection metabolic status and prior HbA1c measurements were unavailable, and HbA1c during acute illness may underestimate pre-existing dysglycaemia. Additionally, residual confounding from factors such as corticosteroid exposure or unmeasured metabolic variables cannot be completely ruled out. Although corticosteroid use is a known confounder for hyperglycaemia, it was not identified as an independent predictor of mortality or ICU admission in our cohort. This is likely because corticosteroid administration was strictly protocol-driven based on the presence of hypoxaemia. Therefore, admission SpO_2_, which was included in our model, served as the primary marker of disease severity, rendering the statistical contribution of corticosteroid therapy redundant in the predictive analysis. Finally, as an observational study, causal relationships between cytokine activation, insulin resistance, and clinical outcomes cannot be definitively established. The study was designed to identify associations and prognostic patterns, not to establish causal pathways. In addition, although selected cytokines were associated with adverse outcomes, the study was not specifically designed or powered to formally test the incremental predictive performance of cytokine profiling beyond routine clinical and metabolic markers (e.g., admission SpO_2_, glucose, and HOMA-IR).

Despite these limitations, the study provides valuable insights into the metabolic and inflammatory alterations associated with COVID-19 and underscores the importance of early glycaemic assessment in patients without known diabetes.

## Figures and Tables

**Table 1:** Admission metabolic markers and cytokine comparisons.

Variable	Normoglycaemia	Dysglycaemia	*p*-Value	Stress Hyperglycaemia	Newly Diagnosed	*p*-Value
	(*n* = 72)	(*n* = 72)		(*n* = 43)	Diabetes (*n* = 29)	
**Metabolic markers**						
HbA1c (mmol/mol)	28.0 (21.5–35.0)	36.0 (29.0–44.0)	0.03	29.0 (25.0–44.0)	36.5 (29.0–44.0)	0.24
HbA1c (%)	4.7 (4.2–5.0)	5.5 (4.8–6.2)	0.01	5.1 (4.2–6.2)	5.5 (5.1–6.3)	0.12
Insulin ^†^	8.4 (6.8–11.7)	18.5 (13.9–22.1)	<0.001	17.3 (12.3–21.8)	19.7 (16.5–24.2)	0.08
HOMA-IR	1.9 (1.5–2.7)	6.8 (5.1–12.8)	<0.001	5.6 (4.5–8.0)	11.2 (6.6–15.5)	<0.001
**Cytokines (pg/mL)**						
IL-6	62.5 (22.9–126.0)	121.8 (57.9–231.5)	0.001	112.9 (58.0–177.9)	172.3 (26.4–426.6)	0.43
IL-8	19.7 (12.2–32.0)	50.9 (22.1–87.8)	<0.001	51.2 (22.4–75.4)	49.5 (15.8–95.8)	0.70
IL-2	3.6 (2.1–5.4)	4.9 (2.3–12.8)	0.008	4.8 (2.3–12.4)	4.9 (2.2–13.1)	0.95
IL-4	2.1 (0.8–3.2)	3.0 (0.7–4.3)	0.10	3.3 (1.2–4.4)	1.0 (0.6–4.3)	0.08
IFN-γ	2.3 (1.8–3.4)	3.04 (2.2–4.5)	0.004	3.3 (2.3–4.3)	2.8 (2.1–4.7)	0.36
TNF-α	0.6 (0.4–0.9)	1.4 (0.6–3.2)	<0.001	2.0 (0.9–3.5)	1.0 (0.6–2.5)	0.08

**Notes:** Values are shown as median (IQR). Statistical comparisons performed using Mann–Whitney U test. HbA1c is expressed in both mmol/mol (IFCC) and % (NGSP/DCCT) units. ^†^ Insulin expressed in μIU/mL. Fasting glucose used for HOMA-IR calculation is expressed in mmol/L; HOMA-IR was calculated as fasting insulin (μIU/mL) × fasting glucose (mmol/L)/22.5. At admission, dysglycaemic patients demonstrated significantly higher metabolic dysfunction (elevated HbA1c, insulin, and HOMA-IR) alongside an amplified pro-inflammatory cytokine signature. Within the dysglycaemic cohort, HOMA-IR was significantly higher in newly diagnosed diabetes compared with stress hyperglycaemia, indicating greater underlying insulin resistance, whereas cytokine concentrations were comparable between subgroups (no statistically significant differences for any cytokine in the SHG vs. NOD comparison). **Abbreviations:** IQR, interquartile range; HbA1c, glycated haemoglobin; HOMA-IR, Homeostatic Model Assessment for Insulin Resistance; IL, interleukin; IFN-γ, interferon gamma; TNF-α, tumour necrosis factor alpha; SHG, stress hyperglycaemia; NOD, newly diagnosed diabetes.

**Table 2:** Admission cytokine concentrations stratified by in-hospital outcome (survivors vs. non-survivors).

Cytokine (pg/mL)	Survivors (*n* = 94)	Non-Survivors (*n* = 50)	*p*-Value
IL-6	63.5 (24.7–142.0)	122.7 (61.6–197.1)	0.009
IL-8/CXCL8	22.7 (12.5–47.7)	46.3 (20.6–81.1)	0.01
TNF-α	0.67 (0.5–1.6)	0.99 (0.5–2.5)	0.15
IFN-γ	2.8 (1.9–4.1)	2.9 (2.1–3.8)	0.52
IL-2	4.3 (2.1–8.1)	4.6 (2.0–12.9)	0.28
IL-4	1.9 (0.7–3.4)	3.2 (1.2–4.1)	0.08

**Notes:** Data are presented as median (IQR); concentrations expressed in pg/mL. P-values by Mann–Whitney U test. Non-survivors demonstrated significantly elevated admission concentrations of IL-6 and IL-8/CXCL8 compared with survivors, whilst other cytokines showed no significant differences between outcome groups. **Abbreviations:** IQR, interquartile range; IL, interleukin; IFN-γ, interferon gamma; TNF-α, tumour necrosis factor alpha; CXCL8, C-X-C Motif Chemokine Ligand 8 (also known as IL-8).

**Table 3:** ROC curve analysis for prediction of ICU admission, prolonged hospitalisation, and mortality.

Outcome/Variable	AUC	95% CI	Sensitivity (%)	Specificity (%)	Cut-Off	Youden Index	*p*-Value
**ICU admission**							
Length of hospitalisation (days)	0.670	0.586–0.746	59.5	70.1	>10	0.296	0.001
BMI (kg/m^2^)	0.615	0.530–0.695	51.4	72.9	>26.99	0.243	0.03
LDH at follow-up (U/L)	0.824	0.751–0.882	70.5	86.5	>341	0.569	<0.001
CRP at follow-up (mg/L)	0.739	0.659–0.808	91.9	44.9	>26	0.368	<0.001
**Prolonged hospitalisation (>10 days)**							
BMI (kg/m^2^)	0.641	0.556–0.719	50.0	72.9	>26.23	0.230	0.002
Haematocrit at follow-up	0.647	0.563–0.724	68.9	62.9	>0.388	0.318	0.002
**Mortality**							
Age (years)	0.671	0.588–0.747	64.0	62.8	>75	0.267	<0.001
SpO_2_ at admission (%)	0.685	0.603–0.760	64.0	64.9	≤87	0.289	<0.001
LDH at follow-up (U/L)	0.773	0.695–0.839	66.0	85.9	>393	0.519	<0.001
CRP at follow-up (mg/L)	0.767	0.690–0.834	62.0	81.9	>85	0.439	<0.001

**Notes:** ROC analysis was performed to determine the optimal cut-off values for predicting clinical outcomes. The Youden index (sensitivity + specificity − 1) was used to identify the optimal threshold. Variables were selected based on bivariate significance or clinical plausibility; only those that remained statistically significant predictors in the multivariable logistic regression model for their respective outcome are presented. Variables significant for one outcome (e.g., prolonged hospitalisation) are not necessarily significant predictors of another outcome (e.g., mortality) and should not be interpreted as such. **Abbreviations:** AUC, area under the curve; CI, confidence interval; BMI, body mass index; LDH, lactate dehydrogenase; CRP, C-reactive protein; SpO_2_, peripheral oxygen saturation; ICU, intensive care unit.

**Table 4:** Baseline demographic characteristics and comorbidities by admission glycaemic status.

Characteristic	Normoglycaemia (*n* = 72)	Dysglycaemia (*n* = 72)	*p*-Value
**Demographics**			
Age (years), median (IQR)	76 (62–84)	74 (64–81)	0.64
Sex, *n* (%)			
Male	33 (45.8)	38 (52.8)	0.41
Female	39 (54.2)	34 (47.2)	0.41
Weight (kg), median (IQR)	75 (66–83)	80 (70–90)	0.05
BMI (kg/m^2^), median (IQR)	24.87 (22.87–28.05)	25.00 (23.28–28.00)	0.55
Day of illness at admission, median (IQR)	7 (4–11)	8 (6–10)	0.13
Length of stay (days), median (IQR)	8 (6–12)	10 (8–14)	0.005
Vaccinated, *n* (%)	35 (48.6)	25 (34.7)	0.09
**Comorbidities,** ***n*** **(%)**			
Arterial hypertension	51 (70.8)	55 (76.4)	0.45
Cardiomyopathy	19 (26.4)	18 (25.0)	0.85
Atrial fibrillation	10 (13.9)	9 (12.5)	0.81
COPD	5 (6.9)	5 (6.9)	>0.99
Asthma	1 (1.4)	4 (5.6)	0.37
Malignancy	7 (9.7)	4 (5.6)	0.35
Chronic kidney disease	7 (9.7)	3 (4.2)	0.19
Prior stroke (CVI)	6 (8.3)	10 (13.9)	0.29
Psychiatric disorders	7 (9.7)	8 (11.1)	>0.99

**Notes:** Values are shown as median (IQR) for continuous variables and *n* (%) for categorical variables. Group differences evaluated using Mann–Whitney U test for continuous variables and $\chi^{2}$ test or Fisher’s exact test for categorical variables, as appropriate. The dysglycaemia group comprises stress hyperglycaemia (*n* = 43; 59.7%) and newly diagnosed diabetes (*n* = 29; 40.3%). No statistically significant differences in baseline demographic characteristics or comorbidities were observed between groups, except for length of hospital stay (*p* = 0.005). **Abbreviations:** IQR, interquartile range; BMI, body mass index; COPD, chronic obstructive pulmonary disease; CVI, cerebrovascular insult.

## Data Availability

The data presented in this study are available within the article. Additional data supporting the findings of this study are available from the corresponding author upon reasonable request, subject to consideration of ethical and privacy issues. This research forms part of the first author’s doctoral dissertation, and related datasets will be published in subsequent publications.

## Abbreviations and Symbols

The following abbreviations and symbols are used in this manuscript (in alphabetical order):

25(OH)D	25-Hydroxyvitamin D (Calcidiol)
ACE2	Angiotensin-Converting Enzyme 2
ADA	American Diabetes Association
ALP	Alkaline Phosphatase
ALT	Alanine Aminotransferase
ANOVA	Analysis of Variance
aPTT	Activated Partial Thromboplastin Time
AST	Aspartate Aminotransferase
AUC	Area Under the Curve
BMI	Body Mass Index
CI	Confidence Interval
CK	Creatine Kinase
CLIA	Chemiluminescent Immunoassay
COPD	Chronic Obstructive Pulmonary Disease
COVID-19	Coronavirus Disease 2019
CRP	C-Reactive Protein
CVI	Cerebrovascular Insult
CXCL8	C-X-C Motif Chemokine Ligand 8 (also known as IL-8)
DCCT	Diabetes Control and Complications Trial
D-dimer	Fibrin Degradation Product
ECDC	European Centre for Disease Prevention and Control
ECLIA	Electrochemiluminescence Immunoassay
GGT	Gamma-Glutamyl Transferase
HbA1c	Glycated Haemoglobin
HFNC	High-Flow Nasal Cannula
HOMA-IR	Homeostatic Model Assessment for Insulin Resistance
hs-TnI	High-Sensitivity Troponin I
ICU	Intensive Care Unit
IFCC	International Federation of Clinical Chemistry
IFN-γ	Interferon Gamma
IL	Interleukin (e.g., IL-2, IL-4, IL-6, IL-8)
IMV	Invasive Mechanical Ventilation
IQR	Interquartile Range
LDH	Lactate Dehydrogenase
LOCI	Luminescent Oxygen Channelling Immunoassay
NGSP	National Glycohemoglobin Standardisation Program
NOD	Newly Diagnosed Diabetes
OR	Odds Ratio
PCT	Procalcitonin
PT	Prothrombin Time
RDW	Red Cell Distribution Width
ROC	Receiver Operating Characteristic
RT-PCR	Reverse Transcription Polymerase Chain Reaction
SARS-CoV-2	Severe Acute Respiratory Syndrome Coronavirus 2
SHG	Stress Hyperglycaemia
SpO_2_	Peripheral Oxygen Saturation
STROBE	Strengthening the Reporting of Observational Studies in Epidemiology
Th1	T Helper 1 Cell (pro-inflammatory lymphocyte subtype)
Th2	T Helper 2 Cell (regulatory/anti-inflammatory lymphocyte subtype)
TNF-α	Tumour Necrosis Factor Alpha
α	Significance Level (Type I Error Rate)
β-cells	Beta Cells (Pancreatic Insulin-Producing Cells)
$\chi^{2}$	Chi-Square Test
*n*	Sample size
*p*	Probability value (*p*-Value)

## Appendix A

**Table A1:** Metabolic markers and cytokine concentrations by ICU transfer status.

Variable	No ICU (n = 107)	ICU (n = 37)	*p*-Value
	Median (IQR)	Median (IQR)	
**Metabolic markers**			
HbA1c (mmol/mol)	36.0 (29.0–41.0)	28.5 (24.5–44.0)	0.41
HbA1c (%)	5.4 (4.8–5.8)	5.0 (4.2–6.2)	0.47
Insulin (μIU/mL)	11.2 (7.1–17.3)	18.3 (9.9–21.2)	0.56
HOMA-IR	3.0 (1.8–6.7)	5.2 (3.0–9.1)	0.04
**Cytokines (pg/mL)**			
IL-6	65.0 (25.1–177.9)	98.0 (60.6–167.5)	0.28
IL-8 (CXCL8)	23.1 (12.5–50.5)	50.3 (21.6–71.7)	0.03
IL-2	4.3 (2.5–8.8)	3.7 (1.8–9.3)	0.44
IL-4	2.1 (0.8–3.8)	2.1 (0.6–3.4)	0.60
IFN-γ	2.7 (1.9–3.6)	3.1 (2.3–4.5)	0.04
TNF-α	0.7 (0.5–1.7)	1.0 (0.5–2.6)	0.29

**Notes:** Values are shown as median (IQR). Statistical comparison performed using Mann–Whitney U test. Patients requiring ICU transfer had significantly higher HOMA-IR (*p* = 0.04), IL-8/CXCL8 (*p* = 0.03), and IFN-γ (*p* = 0.04) concentrations compared with those managed on the general ward, whilst other metabolic markers and cytokines did not differ significantly between groups. **Abbreviations:** ICU, intensive care unit; IQR, interquartile range; HbA1c, glycated haemoglobin; HOMA-IR, Homeostatic Model Assessment for Insulin Resistance; IL, interleukin; IFN-γ, interferon gamma; TNF-α, tumour necrosis factor alpha; CXCL8, C-X-C Motif Chemokine Ligand 8 (also known as IL-8).

**Table A2:** Admission oxygenation, respiratory support, severity, and in-hospital outcomes by glycaemic status.

Variable	Normoglycaemia (*n* = 72)	Dysglycaemia (*n* = 72)	*p*-Value
**Radiological findings**			
Pneumonia, *n* (%)	64 (88.8)	72 (100)	0.003
Unilateral, *n* (%)	11 (15.3)	6 (8.3)	0.200
Bilateral, *n* (%)	53 (74.6)	66 (91.7)	0.006
**Oxygenation and respiratory support**			
Admission SpO_2_, (%), median (IQR)	90 (86–96)	87 (82–90)	<0.001
Oxygen supplementation O_2_, *n* (%)	56 (77.8)	69 (97.2)	<0.001
High-flow nasal cannula (HFNC), *n* (%)	13 (18.1)	37 (51.4)	<0.001
Invasive mechanical ventilation (IMV), *n* (%)	7 (9.7)	24 (33.3)	0.001
**Disease severity**			
Critical COVID-19, *n* (%)	8 (11.1)	22 (30.6)	0.004

**Notes:** Values are shown as median (IQR) for continuous variables and *n* (%) for categorical variables. Statistical comparisons performed using Mann–Whitney U test for continuous variables and *χ*^2^ test or Fisher’s exact test for categorical variables. All patients in the dysglycaemic group had radiologically confirmed pneumonia, with bilateral pulmonary involvement significantly more frequent than in the normoglycaemic group (91.7% vs. 74.6%; *p* = 0.006). Dysglycaemic patients also demonstrated significantly greater requirements for supplemental oxygen, high-flow nasal cannula, and invasive mechanical ventilation, as well as a higher rate of critical COVID-19, compared with normoglycaemic controls. **Abbreviations:** SpO_2_, peripheral oxygen saturation; IQR, interquartile range; HFNC, high-flow nasal cannula; IMV, invasive mechanical ventilation.

**Table A3:** Exploratory non-glycaemic biomarkers at admission stratified by in-hospital outcome.

Biomarker	Survivors (*n* = 94)	Non-Survivors (*n* = 50)	*p*-Value
25(OH)D (nmol/L)	31.5 (23.2–45.6)	19.9 (13.3–29.0)	<0.001
RDW (%)	13.4 (12.9–14.0)	14.6 (13.7–15.9)	0.001

**Notes:** Data are presented as median (IQR). *p*-values by Mann–Whitney U test. These analyses were exploratory and were not pre-specified primary or secondary endpoints; findings should therefore be interpreted as hypothesis-generating only. Non-survivors exhibited significantly lower 25(OH)D concentrations and higher RDW values at admission compared with survivors, consistent with prior reports linking vitamin D deficiency and elevated RDW to adverse COVID-19 outcomes. **Abbreviations:** IQR, interquartile range; 25(OH)D, 25-hydroxyvitamin D (calcidiol); RDW, red cell distribution width.
